# A new algorithm to find fuzzy Hamilton cycle in a fuzzy network using adjacency matrix and minimum vertex degree

**DOI:** 10.1186/s40064-016-3473-x

**Published:** 2016-10-22

**Authors:** A. Nagoor Gani, S. R. Latha

**Affiliations:** 1 PG and Research Department of Mathematics, Jamal Mohamed College (Autonomous), Tiruchirappalli, Tamil Nadu 620020 India; 2 Department of Mathematics, Sona College of Technology (Autonomous), Salem, Tamil Nadu 636 005 India

**Keywords:** Fuzzy graph, Degree of a vertex in a fuzzy graph, Fuzzy Hamiltonian path, Fuzzy Hamiltonian cycle, Adjacency matrix, 05C72, 05C85, 05C38

## Abstract

A Hamiltonian cycle in a graph is a cycle that visits each node/vertex exactly once. A graph containing a Hamiltonian cycle is called a Hamiltonian graph. There have been several researches to find the number of Hamiltonian cycles of a Hamilton graph. As the number of vertices and edges grow, it becomes very difficult to keep track of all the different ways through which the vertices are connected. Hence, analysis of large graphs can be efficiently done with the assistance of a computer system that interprets graphs as matrices. And, of course, a good and well written algorithm will expedite the analysis even faster. The most convenient way to quickly test whether there is an edge between two vertices is to represent graphs using adjacent matrices. In this paper, a new algorithm is proposed to find fuzzy Hamiltonian cycle using adjacency matrix and the degree of the vertices of a fuzzy graph. A fuzzy graph structure is also modeled to illustrate the proposed algorithms with the selected air network of Indigo airlines.

## Background

The ideas of Graph theory are highly utilized by computer science applications and research areas such as data mining, image segmentation, clustering, image capturing, networking, etc. Modeling of network topologies can be done using graph concepts. Paths, walks, and circuits in graph theory are used in tremendous applications like traveling salesman problem, database design concepts and resource networking. This leads to the development of new algorithms and new theorems.

A graph is a pictorial representation for a set of objects in which some pairs are connected by links. The interconnected objects are represented by points called as vertices and the links that connect the vertices are called edges. Two vertices connected by an edge are said to be adjacent. An example of a graph is the route map that roadways produce. Here the vertices are the cities to which a bus runs and the roads are the edges that connect two vertices. The relationships between interconnected computers in a network follow the principles of graph theory. For instance, the molecular and chemical structure of a substance, the DNA structure of an organism, etc., are represented by graphs.

One of the usages of graph theory is to give a unified formulary for varying distinct problems and then suffices algorithms to come up with the best solution. This has led to the birth of a special class of algorithms, the so–called graph algorithms. In many cases, however, some aspects of a graph theoretic problem may be uncertain. For example, a vehicle’s travel time or its capacity on a road may not be determined accurately. In such cases, it is very common to use fuzzy logic methods to deal with the uncertainty.

The concept of fuzzy sets and fuzzy relations was introduced by Zadeh (1965) in 1965 and further studied in Zadeh (2005). A fuzzy set of a base set $V$ is specified by its membership function $\sigma ,$ where $\sigma :V \to \left[ {0,1} \right]$ assigning to each $u \in V$ the degree or grade to which $u$ belongs to $\sigma$. It was Rosenfeld (1975) who considered fuzzy relations on fuzzy sets and developed the theory of fuzzy graphs in 1975. During the same time, Yeh and Bang (1975) have also introduced various connectedness concepts in fuzzy graphs. After the pioneering work of Rosenfeld (1975) and Yeh and Bang (1975) in 1975, several positive outcomes were found with deeper results. Also fuzzy analogues of many other graphically theoretic concepts that include cycles and co cycles of fuzzy graphs Mordeson and Nair (1996) were developed. Zadeh (1968, 2008) introduced a basic concept which was fuzzy rather than precise in nature, eventually prove to be useful in a wide variety of problems relating to information processing, control, pattern recognition, system identification, artificial intelligence and more generally, decision-making processes involving incomplete or uncertain data. The concept is called fuzzy algorithm. Fuzzy logic is not ‘fuzzy’. It is a precise logic of imprecision and approximate reasoning. Imprecision is meant in the sense of vagueness. Fuzzy set theory provides a strict mathematical framework in which vague conceptual phenomena can be precisely and rigorously studied Zimmermann (2010). Fuzzy sets are visualized by fuzzy graph theory. The concept of connectivity plays an important role in both theory and applications of fuzzy graphs. Bhattacharya (1987) has established some connectivity concepts regarding fuzzy cut nodes and fuzzy bridges.

A Hamiltonian cycle, also called a Hamiltonian circuit, Hamilton cycle or Hamilton circuit is a graph cycle through a graph that visits each node exactly once Skiena (1990). A graph possessing a Hamiltonian cycle is said to be a Hamiltonian graph. The Hamiltonian cycle is named after Sir William Rowan Hamilton, who devised a puzzle which resolved into a path along the polyhedron edges of a dodecahedron. There are n! different sequence of vertices that might be Hamiltonian paths in a given n-vertex graph. Testing all possible sequences would be very slow and tedious. Determining if a graph is Hamiltonian is well known to be NP-complete Karp (1972). So a single most efficient algorithm is not known. The only known way to determine whether a given general graph has a Hamiltonian cycle is to undertake an at most exhaustive search.

## Related work

A number of sufficient conditions for a connected simple graph G of order n to be Hamiltonian have been proved. In 1952, Dirac (1952) derived some relations between the degree of the nodes in a graph and the lengths of the circuits contained in it. A graph with sufficiently many edges must contain a Hamiltonian cycle. This sufficient condition for a graph to be Hamiltonian was given by Ore (1960). He proved that in a graph, if the sum of the degrees of every pair of non-adjacent vertices has a sum that at least equals the total number of vertices in the graph, then the graph is Hamiltonian. The relationship between the circumference of a graph and its order, size and vertex degrees was examined by Bondy (1971). Chvatal (1972) provided the best possible generalizations of the theorems of Dirac (1952), Posa (1962) and Bondy (1969) that gave successively weaker sufficient conditions for a graph to be Hamiltonian. Also, he deduced some corollaries on Hamiltonian paths, n—Hamiltonian graphs and Hamiltonian bipartite graphs. During the same year Chvatal and Erdos (1972) proved that, if G is a graph with at least three vertices, for some s, G is s-connected and contains no independent set of more than s vertices, then G has a Hamiltonian circuit. A new sufficient condition for a graph to be Hamiltonian was given by Fan (1984) that does not require that the closure of the graph should be complete and so it is independent of the conditions given by Bondy (1971) and Chvatal (1972). Chen (1993) proved that if G is 2—connected graph and ${ \hbox{max} }\left\{ {d\left( u \right),d\left( v \right)} \right\} \ge n/2$, for each pair of non-adjacent vertices $u,v$ with $1 \le \left| {N\left( u \right) \cap N\left( v \right)} \right| \le \alpha - 1$, then G is Hamiltonian, where $\alpha$ is the independence number of a graph G with order n. This result generalized Fan’s (1984) result on Hamiltonian graphs. In 1996, Chen et al. (1996) proved that if every essential independent set S of order $k \ge 2$ in a $k -$ connected graph of order p satisfied ${ \hbox{max} }\left\{ {d\left( v \right)/v \in S} \right\} \ge p/2$, then G is Hamiltonian (an independent set S of a graph G is said to be an essential independent set of a graph G if S has a pair of vertices distance two apart). Rahman and Kaykobad (2005) gave better conditions than that provided by Ore for the existence of Hamiltonian paths in a graph. Zhao et al. (2007) consolidated and extended the theorems of Dirac (1952), Ore (1960), Fan (1984), Chen (1993), Chen et al. (1996).

Hamiltonicity of random graphs was studied by Lee and Sudakov (2012). Shang (2015) proved that if $p \gg \ln n/n$, then every subgraph of random bipartite graph $G\left( {n,n,p} \right)$ with minimum degree at least $\left( {\frac{1}{2} + o\left( 1 \right)} \right)np$ is Hamiltonian. The proof uses Posa’s (1976) rotation and extension method and is closely related to a recent work of Lee and Sudakov (2012). Dudek et al. (2012) studied the existence of properly colored and rainbow Hamilton cycles in colored k-uniform complete hypergraphs, $k \ge 3$. Rahman et al. (2014) presented a new degree based sufficient conditions for the existence of Hamiltonian paths in a graph. In 2015, Dudek and Ferrara (2015) revised the results proved by Dudek, Frieze, and Rucinski (2012) and showed that there is a constant $c^{\prime} = c^{\prime}\left( {k,l} \right)$ such that every $\left( {l,c_{n}^{{{\prime }k - l}} } \right)$—bounded edge—colored $K_{n}^{\left( k \right)}$ contains a properly colored $l -$ overlapping Hamilton cycle.

In 1974, Rubin (1974) gave a search procedure that would determine whether Hamilton paths or circuits exist in a given graph and would find one or all of them. The search procedure divides the edges of the graph into three classes: those that must be in the path, those that cannot be in the path, and undecided. As the search proceeds, a set of decision rules classified the undecided edges, and determined whether to halt or continue the search. The importance of Hamiltonian graphs has been found in the case of the traveling salesman problem when the graph is a weighted graph. Several works have been done to find the number of Hamiltonian cycles in a Hamiltonian graph. As the number of vertices and edges grow, it becomes harder to keep track of all the different ways the vertices are connected. Analysis of large graphs often requires computer assistance. So it is necessary to express graphs through matrices.

An adjacency matrix is a square matrix that is used to represent a finite graph. The elements of the matrix indicate whether the pairs of vertices are adjacent or not in the graph. With an adjacency matrix, testing the existence of an edge between two vertices can be determined at once. In this paper, we have developed another algorithm to find fuzzy Hamiltonian cycle using adjacency matrix of a fuzzy graph. In “Preliminaries” section basic definitions and theorems are presented. In “Algorithm to find fuzzy Hamiltonian cycle in a fuzzy graph G” section, two algorithms, one is using the adjacency matrix of a given fuzzy graph and the other is using the minimum vertex degree of a fuzzy graph are proposed. The proposed algorithms are illustrated with the route map of Indigo Airlines Prabir De (2015). A copy of the route map is given in Fig. 2. Here the vertices are the cities to which Indigo Airlines fly and two vertices are connected if a direct flight flies between them and the paper is concluded in “Conclusion”. We consider simple and undirected fuzzy graph.

## Preliminaries

Basic definitions are presented in this section.

### **Definition 1** Nagoor Gani and Chandrasekaran (2010)

A fuzzy graph is a pair of functions σ: V → [0, 1] and μ: V × V → [0, 1], where for all *u, v* in V we have μ (*u, v*) ≤ σ (*u*) Λ σ (*v*).

### **Definition 2** Nagoor Gani and Chandrasekaran (2010)

Let $G:\left( {\sigma ,\mu } \right)$ be a fuzzy graph. The degree of a vertex u is $d_{G} \left( u \right) = d\left( u \right) = \mathop \sum \nolimits_{u \ne v} \mu \left( {u,v} \right)$.

### **Definition 3**

If $$ G:\left( {\sigma ,\mu } \right) $$ is a fuzzy graph then its adjacency matrix is defined as $$ X $$ where $$ X_{ij} = \mu \left( {v_{i} ,v_{j} } \right) $$ for $$ i \ne j $$ & when $$ i = j, $$ $$ X_{ii} = \sigma \left( {v_{i} } \right), $$ if the fuzzy relation is reflexive and $$ X_{ii} = 0 $$, if the fuzzy relation is not reflexive.

In this paper, fuzzy relation which is not reflexive is considered. Hence the adjacency matrix $A\left( G \right)$ of $G$ is an n x n symmetric matrix with zero diagonal. The degree of the vertices of $G$ represented by adjacency matrix is the row sums of $A\left( G \right)$. Every non-zero entry in $A\left( G \right)$ represents the edge weight $e_{ij} = \mu \left( {u_{i} ,u_{j} } \right)$ between two vertices $u_{i}$ and $u_{j}$.

### **Definition 4**

A path $\rho$ in a fuzzy graph is a sequence of distinct vertices $u_{0} ,u_{1} ,u_{2} , \ldots u_{n}$ such that $\mu \left( {u_{i - 1} ,u_{i} } \right) > 0,1 \le i \le n$; here $n \ge 0$ is called the length of the path $\rho$. The path is called a cycle if $u_{0} = u_{n}$ and $n \ge 3$.

### **Definition 5**

A fuzzy Hamiltonian path is a path that passes through each of the vertices in a fuzzy graph exactly once.

### **Definition 6**

A fuzzy Hamiltonian cycle is a cycle that visits every vertex in a fuzzy graph once with no repeats and being a fuzzy Hamiltonian cycle it must start and end at the same vertex.

### **Proposition 1**

*In a fuzzy graph, if every vertex has exactly two adjacent vertices, then there exists a fuzzy Hamiltonian cycle.*

### *Proof*

Let $G:\left( {\sigma ,\mu } \right)$ be a fuzzy graph with ‘n’ vertices $u_{1} ,u_{2} , \ldots ,u_{n}$ satisfying the hypothesis.

Assume a contradiction. i.e. An edge between any two vertices is removed, say $\mu \left( {u_{1} ,u_{2} } \right)$.

Now, we begin to explore a path starting with (i) the vertex $u_{1} \left( {or u_{2} } \right)$ and it ends with $u_{2} \left( {or u_{1} } \right)$ covering all the vertices of $G$ (or) (ii) any vertex $u_{i}$, either it takes the path $u_{i + 1} ,u_{i + 2} , \ldots ,$ and end with $u_{1}$ or the path $u_{i - 1} ,u_{i - 2} , \ldots ,$ and end with $u_{2}$, excluding some vertices of $G$. Both the cases results with a non-fuzzy Hamiltonian cycle.

## Algorithm to find fuzzy Hamiltonian cycle in a fuzzy graph G

In this section, two algorithms are proposed to find a Fuzzy Hamiltonian cycle in a fuzzy graph.

### Algorithm to find fuzzy Hamiltonian path and fuzzy Hamiltonian cycle in a fuzzy graph G using adjacency matrix of G

**Fig. 1:**
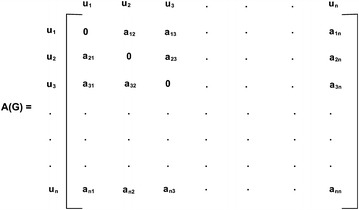
Adjacency matrix of a fuzzy graph of order n

Let $G:\left( {\sigma ,\mu } \right)$ be a fuzzy graph of order n and $A\left( G \right)$ be its adjacency matrix (Fig. 1).

Select a non-zero minimum entry from the adjacency matrix say $a_{ij} = \mu \left( {u_{i} ,u_{j} } \right)$, the edge weight between the vertices $u_{i}$ and $u_{j}$. This gives the initial path that starts with the vertex $u_{i}$ and travels to $u_{j}$. Now in the row of $u_{j}$ select an appropriate non-zero entry and this process is continued until it results in a fuzzy Hamiltonian path which may further extended to find a fuzzy Hamiltonian cycle if exists.

#### Algorithm

- *Step 1*: Form the adjacency matrix $A\left( G \right).$
- *Step 2*: Search for a minimum non-zero entry in $A\left( G \right)$, say $a_{ij}$. (If there are repetitions, then choose any one).
- *Step 3*: If the minimum value chosen does not permit for a fuzzy Hamiltonian path, then the next higher minimum value is selected.
- *Step 4*: Identify the row and column, say $u_{i}$ and $u_{j}$ respectively where the minimum entry appears.
- *Step 5*: Search for a minimum non-zero entry in the row of $$ u_{j} , $$ such that,
  (i) it forms a fuzzy Hamiltonian path. (no repetition of vertices in the path)
  (ii) if the minimum value occurs more than once, then an appropriate entry is selected to get a fuzzy Hamiltonian path.
- *Step 6*: Repeat Step 3 through Step 4 row-wise until a fuzzy Hamiltonian path is found with all n vertices of G. Else conclude, there is no fuzzy Hamiltonian path and go to Step 2 or Step 3 as required.
- If fuzzy Hamiltonian path is true, then at this stage only one row will be left out with no entries marked.
- *Step 7*: Select a non-zero entry from that row to form a fuzzy Hamiltonian cycle, if exists.

The above algorithm is illustrated in the following example.

#### *Example 1*

**Fig. 2:**
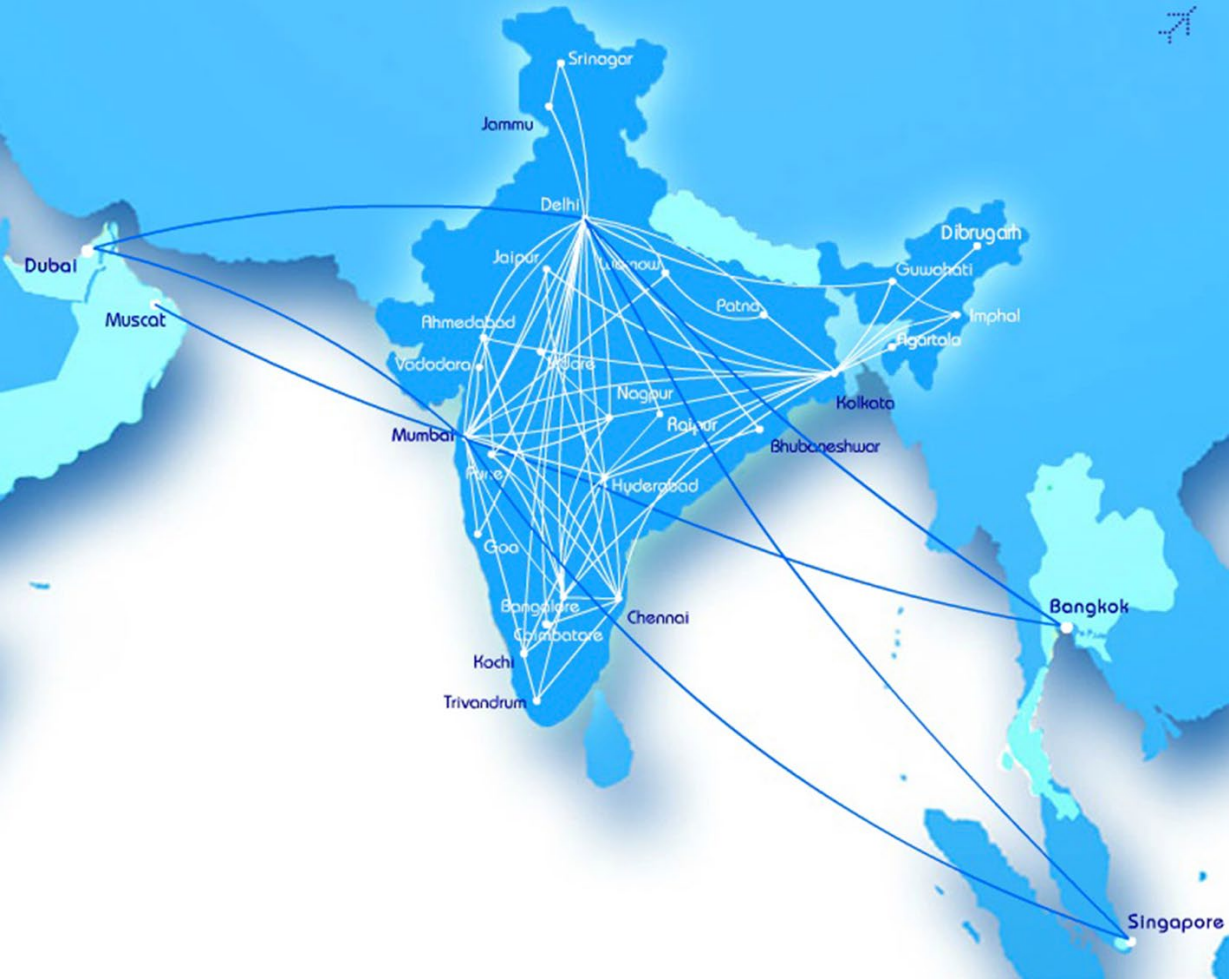
Route map of Indigo Airlines

(Model formulation) A fuzzy graph structure is modeled from the airline network of Indigo airlines (Fig. 2) to illustrate the above algorithm by using the air distances between the cities to find fuzzy Hamiltonian cycle.

**Fig. 3:**
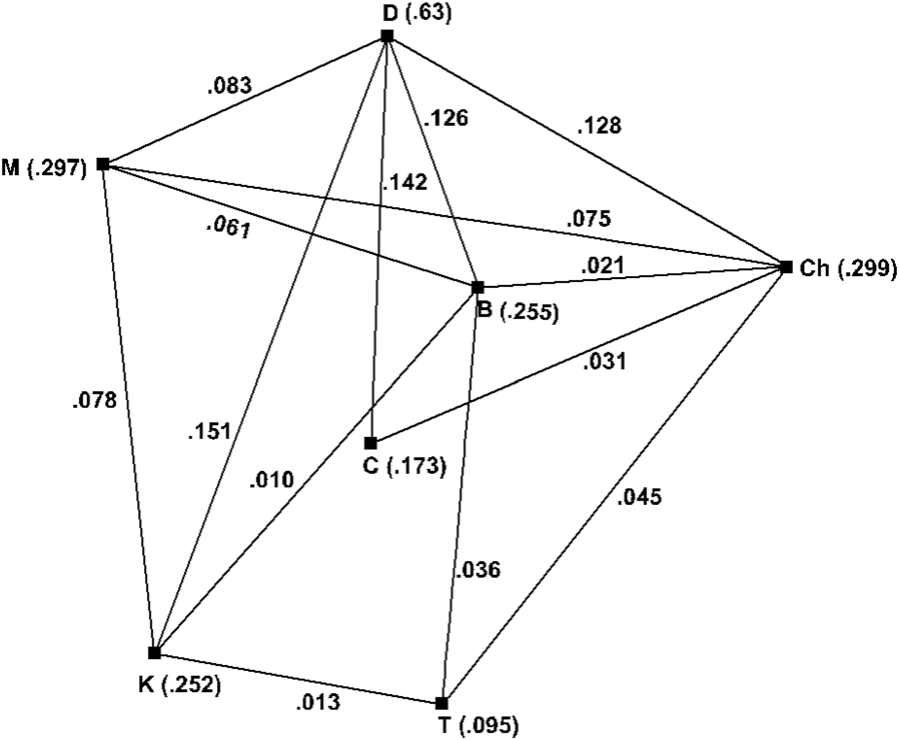
Fuzzy graph of selected cities

From the above map flight routes between the cities Delhi, Mumbai, Bangalore, Chennai, Coimbatore, Kochi and Trivandrum are selected. Figure 3 represents the fuzzy graph of the airline route map in which the vertices denotes the cities Delhi (D), Mumbai (M), Bangalore (B), Chennai (C), Coimbatore (Ch), Kochi (K) and Trivandrum (T). The membership value of every vertex is the ratio of the air distance to all destinations from it to the total air distance of all the air routes and the edge weight is the ratio of the air distance between two cities to the total air distance of all the air routes in the selected network.

**Fig. 4:**
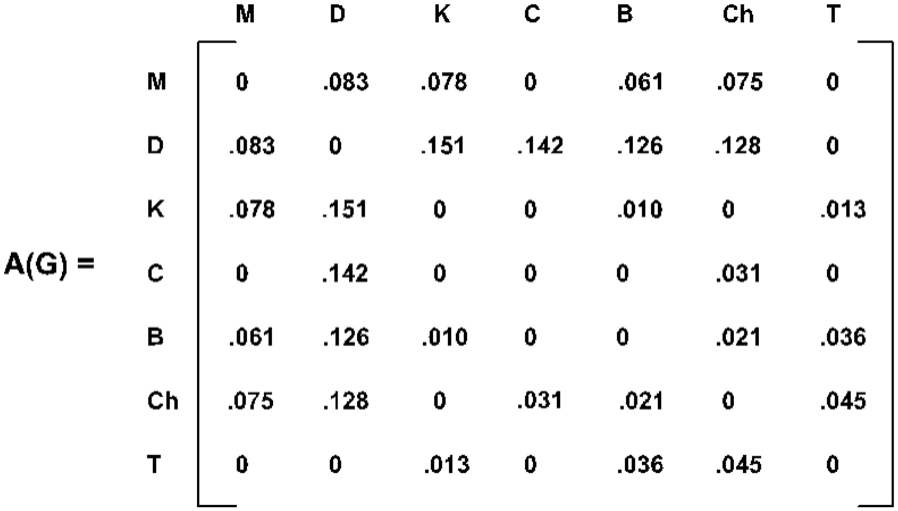
Adjacency matrix of the fuzzy graph of selected cities
- *Step 2*: The minimum non-zero entry appears in the third row and the fifth column of $A\left( G \right)$. Choose the entry in the third row i.e. $a_{35} = .010$.
- *Step 4*: This entry represents the **row** ‘K’ and column ‘**B**’ i.e., from ‘K’ it reaches ‘**B**’. ($$ K - B) $$
- *Step 5*: In the row ‘B’, the minimum non-zero entry is .021 which appears in the column ‘Ch’. i.e., from ‘B’ it goes to ‘Ch’ $(K - B - Ch)$
- *Step 6*: Iteration 1: Repeating Step 3 through Step 4, the path obtained is $(K - B - Ch - C - D - M - K)$, which is rejected as it is a fuzzy circuit.
  - *Iteration 2*: Now, start with the other minimum non-zero entry $$ a_{53} = .010, $$ represented by the row ‘**B**’ and column ‘**K**’ ($$ B - K $$). By algorithm, the path obtained is B $$ - K - T - Ch - C - D - M $$ is a fuzzy Hamiltonian path.
- *Step 7*: At this stage, the row ‘*M*’ is left unmarked. From the row ‘*M*’, select the non-zero entry (which exists) corresponding to the column ‘*B*’, to get a fuzzy Hamiltonian cycle $B - K - T - Ch - C - D - M - B$ (total length = .385).

- *Step 1*: Form the adjacency matrix of $G$ (Fig. 4),

### Minimum vertex degree algorithm to find fuzzy Hamiltonian cycle in a fuzzy graph

This algorithm identifies fuzzy Hamiltonian cycle of a fuzzy graph $G:\left( {\sigma ,\mu } \right)$ using the degrees of the vertices of $G$. It starts with the vertex having the minimum degree and the process is iterated until a fuzzy Hamiltonian cycle is obtained.

#### Algorithm

- *Step 1*: Calculate the degrees of all vertices in G.
- *Step 2*: Start with a vertex of minimum degree. (If there is more than one vertex with same minimum degree, then choose any one).
- *Step 3*: Select a vertex whose degree is next higher to the minimum degree chosen.
- *Step 4*: Identify the adjacent vertices of the minimum vertex degree selected.
- *Step 5*: Reach to the unvisited adjacent vertex of minimum degree. (If more than one adjacent vertex has the same identified minimum degree, then choose any one vertex).
- *Step 6*: Step 4 through Step 5 is repeated until a fuzzy Hamiltonian cycle is found. Else, go to Step 2 or Step 3 as required.

#### *Remark 1*

In the above algorithm step 4 and step 5 shall be repeated starting with each vertex of G to find all possible fuzzy Hamiltonian cycle (if exists). By calculating the length of every fuzzy Hamiltonian cycle, the minimum length covered by fuzzy Hamiltonian cycle(s) can be identified.

Illustration of the above algorithm is explained in the following example.

#### *Example 2*

- *Step 1*: From the fuzzy graph in Fig. 3, the degrees of the vertices of $G$ are $d\left( M \right) = .297; d\left( D \right) = .63;d\left( K \right) = .252;d\left( C \right) = .173;d\left( B \right) = .254;d\left( {Ch} \right) = .3$; $d\left( T \right) = .094$.
- *Step 2*: Select the vertex T, as it has the least degree $(d(T) = .094)$.
- *Step 3*: The adjacent vertices of T are K, B and Ch.
- *Step 4*: From Step 1, $\left( K \right) = .252$, $d\left( B \right) = .254,d\left( {Ch} \right) = .3$ and the minimum of these values is .252 which corresponds to the vertex K. Therefore, the path travels from T to K (T − K)
- *Step 5*: Repeat Steps 3 and 4.
  - *Iteration 1*: The adjacent vertices of K are M, D, T, and B with $d\left( M \right) = .297; d\left( D \right) = .63,$
  - $d\left( T \right) = .094,d\left( B \right) = .254.$ The minimum value is .094 and that corresponds to the vertex T. But, from K, the path cannot move to the vertex T, as it would not form a fuzzy Hamiltonian cycle. Therefore, select the next higher minimum vertex degree B to form the path T $- K - B$.
  - *Iteration 2*: The adjacent vertices of B are D, M, K, T, and Ch with $d\left( D \right) = .63,d\left( M \right) = .297$
  - $d\left( K \right) = .252,d\left( T \right) = .094, d\left( {Ch} \right) = .3.$ The next unvisited vertex with minimum degree is M and the path is T $- K - B - M.$
  - *Iteration 3*: The adjacent vertices of M are D, Ch, B, and K with $d\left( D \right) = .63,d\left( {Ch} \right) = .3,$
  - $d\left( B \right) = .254,d\left( K \right) = .252$. The unvisited vertex with minimum degree is Ch. The path is
  - $$ T - K - B - M - Ch. $$
  - *Iteration 4*: The adjacent vertices of Ch are $d\left( D \right) = .63,d\left( B \right) = .254, d\left( C \right) = .173, d\left( M \right) = .297, d\left( T \right) = .094$ and the path is T $- K - B - M - Ch - C.$
  - *Iteration 5*: The adjacent vertices of C are $d\left( D \right) = .63,d\left( {Ch} \right) = .3$ and the path is
  - $$ T - K - B - M - Ch - C - D. $$
  - *Iteration 6*: The vertices D and T are not connected ⇒ fuzzy Hamiltonian cycle does not exist in the path $T - K - B - M - Ch - C - D.$ Therefore, start with the other minimum vertex degree C and repeat Step 4 and Step 5, the fuzzy Hamiltonian cycle is $C - Ch - T - K - B - M - D - C\left( {total\;length = 0.385} \right).$

#### *Remark 2*

The other possible fuzzy Hamiltonian cycles starting with the next higher minimum degrees are $K - T - B - M - Ch - C - D - K\,\left( {total \;length = .509} \right)$

$$ B - T - K - M - Ch - C - D - B \,\left( {total\; length = .501} \right) $$

$$ M - K - T - B - Ch - C - D - M  \,\left( {total\;length = .404} \right) $$

$$ Ch - T - K - B - M - D - C - Ch \,\left( {total\;length = .385} \right) $$

$$ D - C - Ch - T - K - B - M - D \,\left( {total\;length = .385} \right) $$

## Conclusion

In this context, we consider the adjacency matrix of fuzzy graph to find fuzzy Hamiltonian cycle. When the number of vertices and edges grow higher, it becomes difficult to find fuzzy Hamiltonian cycle and this is made simple using adjacency matrix. The algorithm first finds a fuzzy Hamiltonian path of the fuzzy graph. Once the fuzzy Hamiltonian path is found, then it is easy to complete the algorithm i.e. finding a fuzzy Hamiltonian cycle. Also, a new algorithm is developed by iterating with the minimum vertex degree of a given fuzzy graph to find fuzzy Hamiltonian cycle. From the algorithm, the (approximate) air distance is evaluated from the total length covered by each fuzzy Hamiltonian cycle. A fuzzy graph structure is modeled to illustrate the proposed algorithms with the selected air network of Indigo airlines.
